# Author Correction: Beta activity in human anterior cingulate cortex mediates reward biases

**DOI:** 10.1038/s41467-025-61277-0

**Published:** 2025-06-25

**Authors:** Jiayang Xiao, Joshua A. Adkinson, John Myers, Anusha B. Allawala, Raissa K. Mathura, Victoria Pirtle, Ricardo Najera, Nicole R. Provenza, Eleonora Bartoli, Andrew J. Watrous, Denise Oswalt, Ron Gadot, Adrish Anand, Ben Shofty, Sanjay J. Mathew, Wayne K. Goodman, Nader Pouratian, Xaq Pitkow, Kelly R. Bijanki, Benjamin Hayden, Sameer A. Sheth

**Affiliations:** 1 Department of Neurosurgery, Baylor College of Medicine, Houston, TX 77030 USA; 2 Department of Neuroscience, Baylor College of Medicine, Houston, TX 77030 USA; 3 School of Engineering, Brown University, Providence, RI 02912 USA; 4 Department of Neurosurgery, University of Utah, Salt Lake City, UT 84112 USA; 5 Department of Psychiatry and Behavioral Sciences, Baylor College of Medicine, Houston, TX 77030 USA; 6 Department of Neurological Surgery, UT Southwestern Medical Center, Dallas, TX 75390 USA; 7 Department of Electrical and Computer Engineering, Rice University, Houston, TX 77005 USA; 8 Center for Neuroscience and Artificial Intelligence, Baylor College of Medicine, Houston, TX 77030 USA

**Keywords:** Reward, Psychiatric disorders, Cognitive neuroscience

Correction to: *Nature Communications* 10.1038/s41467-024-49600-7, published online 15 July 2024

In the version of the article initially published, in the Competing interests section, the sentence “W.K.G. has received donated devices from Medtronic and has consulting agreements with Biohaven Pharmaceuticals” was incorrect and has now been amended to “W.K.G receives royalties from Nview, LLC and OCDscales, LLC” in the HTML and PDF versions of the article.

