# Supplementary material for: Beta activity in human anterior cingulate cortex mediates reward biases
Source: Nat Commun. 2024 Jul 15;15:5528. doi: 10.1038/s41467-024-49600-7 (PMC11250824; doi:10.1038/s41467-024-49600-7)
Supplement: Supplementary file 1 — Supplementary Information [file 41467_2024_49600_MOESM1_ESM.pdf]

Beta activity in human anterior cingulate cortex mediates reward biases

Authors: Jiayang Xiao[1,2], Joshua A. Adkinson[1], John Myers[1], Anusha B. Allawala[3], Raissa K. Mathura[1], Victoria Pirtle[1], Ricardo Najera[1], Nicole R Provenza[1], Eleonora Bartoli[1], Andrew J Watrous[1], Denise Oswalt[1], Ron Gadot[1], Adrish Anand[1], Ben Shofty[4], Sanjay J. Mathew[5], Wayne K. Goodman[5], Nader Pouratian[6], Xaq Pitkow[2,7,8], Kelly R. Bijanki[1], Benjamin Hayden[1], Sameer A. Sheth[1,2,5,7]*

Affiliations:

[1]Department of Neurosurgery, Baylor College of Medicine, Houston, TX 77030, United States.

[2]Department of Neuroscience, Baylor College of Medicine, Houston, TX 77030, United States.

[3]School of Engineering, Brown University, Providence, RI 02912, United States.

[4]Department of Neurosurgery, University of Utah, Salt Lake City, UT 84112, United States.

[5]Department of Psychiatry & Behavioral Sciences, Baylor College of Medicine, Houston, TX 77030, United States.

[6]Department of Neurological Surgery, UT Southwestern Medical Center, Dallas, TX 75390, United States.

[7]Department of Electrical and Computer Engineering, Rice University, Houston, TX 77005, United States.

[8]Center for Neuroscience and Artificial Intelligence, Baylor College of Medicine, Houston, TX 77030, United States.

* Corresponding author: Sameer A. Sheth; sasheth@bcm.edu

Supplementary information:

Supplementary Figures 1-2

Supplementary Table 1-2

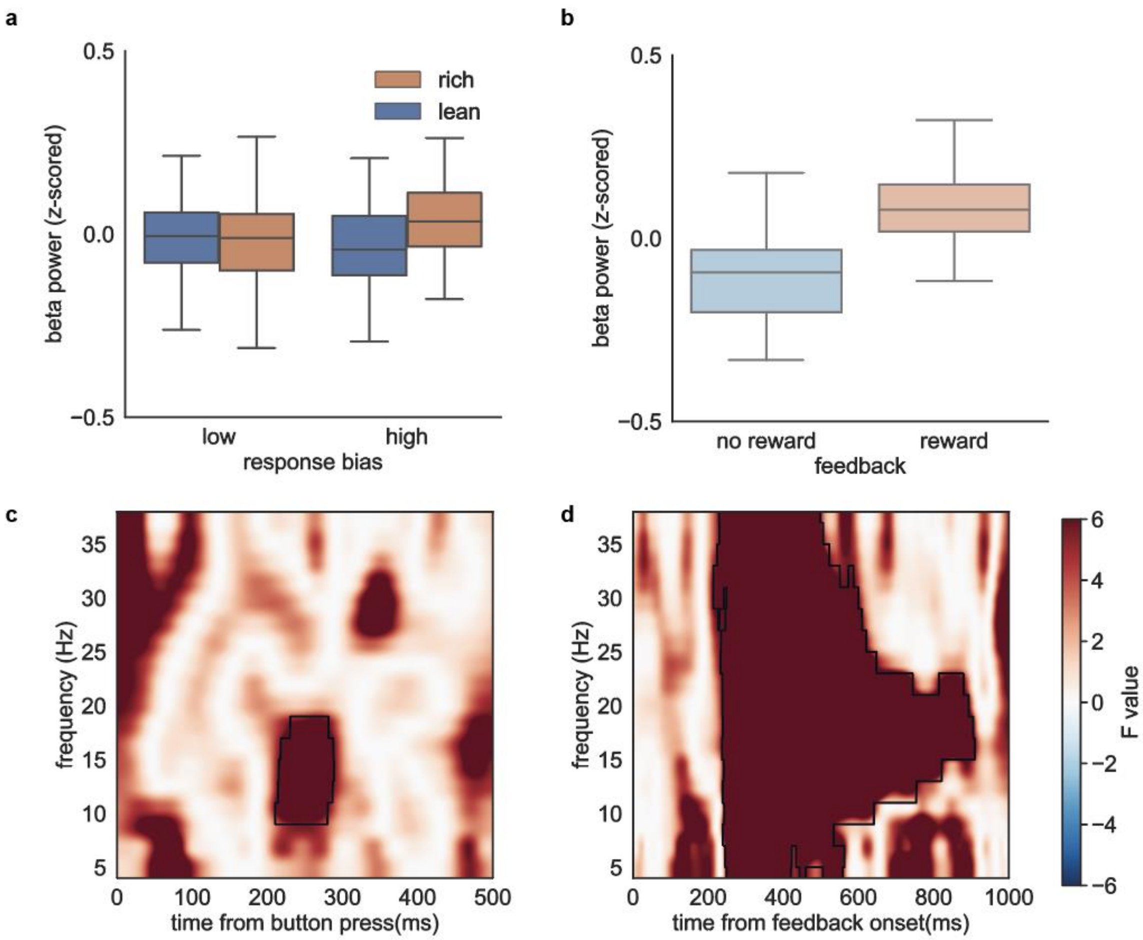

Supplementary Fig. 1: Additional analysis for beta activity. (a) Differences in beta power between the rich and lean trials during the delay period. Boxplots illustrate quartiles at 25% and 75%, with horizontal lines denoting medians, and whiskers extending to 1.5 times the interquartile ranges. n=56 channels for low response bias blocks and n=52 channels for high response bias blocks. Linear mixed model, $p = 0.90$ for the low response bias blocks and $p = 0.0021$ for the high response bias blocks. **(b)** Differences in beta power between the reward and neutral trials during the feedback period. Boxplots illustrate quartiles at 25% and 75%, with horizontal lines denoting medians, and whiskers extending to 1.5 times the interquartile ranges. n=65 channels. Linear mixed model, $p = 2.7*10^{-38}$. **(c)** Time-frequency plot for the difference comparing rich and lean stimulus during the delay period. Red indicates larger power towards rich

stimulus. **(d)** Time-frequency plot for the difference comparing reward and neutral feedback during the feedback period. Red indicates a larger power towards reward feedback.

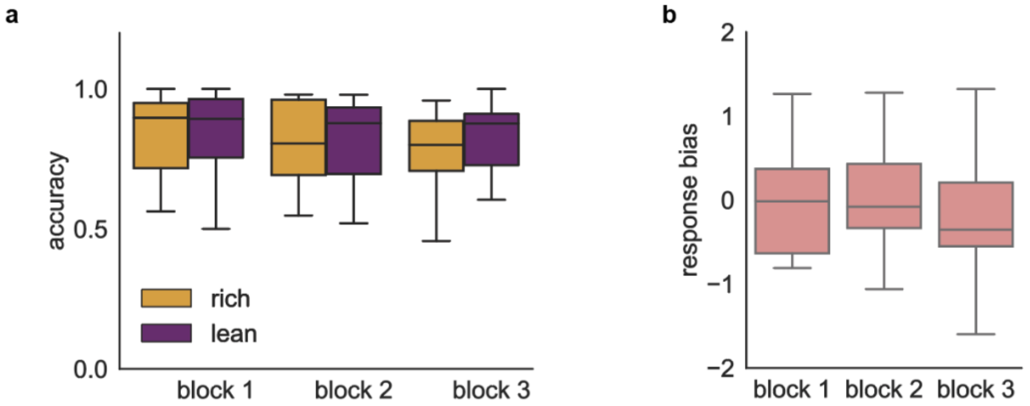

Supplementary Fig. 2: Behavioral performance in depression patients. (a) Accuracy for rich and lean stimuli averaged across all Depression Cohort patients. Boxplots illustrate quartiles at 25% and 75%, with horizontal lines denoting medians, and whiskers extending to 1.5 times the interquartile ranges. n = 12 runs of task. Paired-sample t-test, block1: $p = 0.90$, block2: $p = 0.94$, block3: $p = 0.60$. **(b)** Response bias averaged across all depression patients. A response bias value close to zero indicates there is no preference for choosing the more frequently rewarded stimulus. Boxplots illustrate quartiles at 25% and 75%, with horizontal lines denoting medians, and whiskers extending to 1.5 times the interquartile ranges. n = 12 runs of task. One-sample t test compared with zero, block1: $p = 0.93$, block2: $p = 0.83$, block3: $p = 0.38$.

Supplementary Table. 1.

	ACC	mOFC	lOFC	Amygdala
Epilepsy	65(13)	91(10)	98(10)	42(6)
Depression	36(4)	55(4)	49(4)	31(4)

The numbers outside the parentheses indicate the total number of channels in each of the recording sites. The numbers inside the parentheses indicate the number of patients with electrode coverage in these sites.

Supplementary Table. 2

group	period	freq	coef.	std.err.	t	p	lower bound	upper bound
epilepsy	delay	delta	0.73	8.09	0.09	1	-15.12	16.57
epilepsy	delay	theta	-8.35	4.78	-1.75	0.40	-17.72	1.02
epilepsy	delay	alpha	-2.46	3.55	-0.69	1	-9.43	4.5
epilepsy	delay	gamma	-0.13	1.03	-0.12	1	-2.15	1.9
epilepsy	delay	highgamma	0.49	0.5	0.98	1	-0.49	1.48
epilepsy	feedback	delta	5.13	7.21	0.71	1	-9	19.27
epilepsy	feedback	theta	7.72	4.5	1.72	0.43	-1.1	16.53
epilepsy	feedback	alpha	15.52	3.36	4.62	$\mathbf{2.0*10^{-5}}$	8.93	22.1
epilepsy	feedback	gamma	1.55	0.94	1.66	0.48	-0.28	3.39
epilepsy	feedback	highgamma	0.97	0.45	2.17	0.15	0.09	1.85
depression	delay	delta	4.59	3.56	1.29	0.99	-2.39	11.57
depression	delay	theta	2.82	2.52	1.12	1	-2.12	7.76
depression	delay	alpha	5.02	3.8	1.32	0.94	-2.44	12.47
depression	delay	gamma	-0.97	0.78	-1.24	1	-2.51	0.57
depression	delay	highgamma	-0.24	0.37	-0.64	1	-0.97	0.49
depression	feedback	delta	25.2	3.08	8.18	$\mathbf{1.4*10^{-15}}$	19.16	31.23
depression	feedback	theta	7.97	2.85	2.8	**0.03**	2.39	13.56
depression	feedback	alpha	9.51	3.62	2.63	**0.04**	2.42	16.61
depression	feedback	gamma	2.39	0.68	3.52	**0.002**	1.06	3.72
depression	feedback	highgamma	0.51	0.32	1.58	0.57	-0.12	1.15

Linear mixed effect models used here are two-sided and adjustments are made for multiple comparisons. Bold denotes statistical significance at the $p < 0.05$ level.
