# Supplementary material for: Beta activity in human anterior cingulate cortex mediates reward biases
Source: Nat Commun. 2024 Jul 15;15:5528. doi: 10.1038/s41467-024-49600-7 (PMC11250824; doi:10.1038/s41467-024-49600-7)
Supplement: Supplementary file 3 — Reporting Summary [file 41467_2024_49600_MOESM3_ESM.pdf]

nature portfolio

Corresponding author(s): Sameer Sheth

Last updated by author(s): May 7, 2024

Reporting Summary

Nature Portfolio wishes to improve the reproducibility of the work that we publish. This form provides structure for consistency and transparency in reporting. For further information on Nature Portfolio policies, see our Editorial Policies and the Editorial Policy Checklist.

Statistics

For all statistical analyses, confirm that the following items are present in the figure legend, table legend, main text, or Methods section.

n/a	Confirmed	
☐	☒	The exact sample size (*n*) for each experimental group/condition, given as a discrete number and unit of measurement
☐	☒	A statement on whether measurements were taken from distinct samples or whether the same sample was measured repeatedly
☐	☒	The statistical test(s) used AND whether they are one- or two-sided<br>*Only common tests should be described solely by name; describe more complex techniques in the Methods section.*
☒	☐	A description of all covariates tested
☐	☒	A description of any assumptions or corrections, such as tests of normality and adjustment for multiple comparisons
☐	☒	A full description of the statistical parameters including central tendency (e.g. means) or other basic estimates (e.g. regression coefficient) AND variation (e.g. standard deviation) or associated estimates of uncertainty (e.g. confidence intervals)
☐	☒	For null hypothesis testing, the test statistic (e.g. *F*, *t*, *r*) with confidence intervals, effect sizes, degrees of freedom and *P* value noted<br>*Give P values as exact values whenever suitable.*
☒	☐	For Bayesian analysis, information on the choice of priors and Markov chain Monte Carlo settings
☒	☐	For hierarchical and complex designs, identification of the appropriate level for tests and full reporting of outcomes
☒	☐	Estimates of effect sizes (e.g. Cohen's *d*, Pearson's *r*), indicating how they were calculated

Our web collection on statistics for biologists contains articles on many of the points above.

Software and code

Policy information about availability of computer code

Data collection	We used a Cerebus data acquisition system to collect the data. The behavioral task was implemented in MATLAB using Psychtoolbox-3.
Data analysis	Preprocessing was performed using MATLAB (R2021a). Further analyses were performed using Python 3.7.6. Electrode positions were manually marked using the co-registered CT data in BioImage Suite v3.5b1. Code is available under: https://osf.io/t3usq/.

For manuscripts utilizing custom algorithms or software that are central to the research but not yet described in published literature, software must be made available to editors and reviewers. We strongly encourage code deposition in a community repository (e.g. GitHub). See the Nature Portfolio guidelines for submitting code & software for further information.

Data

Policy information about availability of data

All manuscripts must include a data availability statement. This statement should provide the following information, where applicable:

- Accession codes, unique identifiers, or web links for publicly available datasets
- A description of any restrictions on data availability
- For clinical datasets or third party data, please ensure that the statement adheres to our policy

Processed data is provided at the following address: https://osf.io/t3usq/.The raw intracranial EEG data are available upon request for reasons of patient confidentially. Source data are provided with this paper.

Research involving human participants, their data, or biological material

Policy information about studies with human participants or human data. See also policy information about sex, gender (identity/presentation), and sexual orientation and race, ethnicity and racism.

Reporting on sex and gender	Epilepsy Cohort: fifteen participants (eight males and seven females)<br>Depression Cohort: four participants (two males and two females)<br>Sex and/or gender was considered in the study design. Gender of participants was determined based on self-report.<br>Sex- and gender-based analyses have not been performed due to the limited number of patients and the rarity of opportunities to perform intracranial recordings in this patient population.
Reporting on race, ethnicity, or other socially relevant groupings	These variables were not used in this study.
Population characteristics	Epilepsy Cohort: fifteen participants undergoing invasive monitoring for the treatment of refractory epilepsy (mean age 39 years, range 19-60 years)<br>Depression Cohort: four patients with treatment-resistant depression (mean age 42 years, range 37-58 years)
Recruitment	Epilepsy cohort were recruited at Baylor St. Luke's Medical Center by clinical teams and neurosurgeons. These participants provided verbal and written consent to participate in our study. Participants who were willing to perform behavioral tasks were recruited. Treatment-resistant depression patients were enrolled in an early feasibility trial (NCT03437928). These individuals did not carry significant psychiatric comorbidities based on the trial's exclusion of schizophrenia, bipolar disorder, personality disorders, and neuro-developmental disorders, as these conditions may impact the study results. Additional details regarding the exclusion criteria can be found: https://clinicaltrials.gov/study/NCT03437928#participation-criteria.
Ethics oversight	The study was approved by the Institution Review Board at Baylor College of Medicine.

Note that full information on the approval of the study protocol must also be provided in the manuscript.

Field-specific reporting

Please select the one below that is the best fit for your research. If you are not sure, read the appropriate sections before making your selection.

☒ Life sciences ☐ Behavioural & social sciences ☐ Ecological, evolutionary & environmental sciences

For a reference copy of the document with all sections, see nature.com/documents/nr-reporting-summary-flat.pdf

Life sciences study design

All studies must disclose on these points even when the disclosure is negative.

Sample size	The sample size was determined solely by the availability of patients. Participants who performed the behavioral task were included in the analysis. The resulting sample size was comparable to that of other studies utilizing intracranial recording.
Data exclusions	Channels with excessive noise were excluded to prevent noise from spreading to other channels before any further analysis.
Replication	We did not replicate the present findings in an independent cohort due to the rarity of intracranial recording.
Randomization	The order of stimuli in the task was randomized across participants.
Blinding	Participants were not aware of the goal of this study. Data analysis was not conducted blindly because group information is essential for the analysis.

Reporting for specific materials, systems and methods

We require information from authors about some types of materials, experimental systems and methods used in many studies. Here, indicate whether each material, system or method listed is relevant to your study. If you are not sure if a list item applies to your research, read the appropriate section before selecting a response.

Materials & experimental systems

n/a	Involved in the study
☒	☐ Antibodies
☒	☐ Eukaryotic cell lines
☒	☐ Palaeontology and archaeology
☒	☐ Animals and other organisms
☐	☒ Clinical data
☒	☐ Dual use research of concern
☒	☐ Plants

Methods

n/a	Involved in the study
☒	☐ ChIP-seq
☒	☐ Flow cytometry
☒	☐ MRI-based neuroimaging

Clinical data

Policy information about clinical studies

All manuscripts should comply with the ICMJE guidelines for publication of clinical research and a completed CONSORT checklist must be included with all submissions.

Clinical trial registration	NCT03437928
Study protocol	Protocol may be found at https://www.clinicaltrials.gov/study/NCT03437928
Data collection	Data were collected using a Cerebus data acquisition system at Baylor St. Luke's Medical Center.
Outcomes	Primary outcome is changes in depressive symptoms.
